# Short-term effects of Lumacaftor/Ivacaftor (Orkambi™) on exertional symptoms, exercise performance, and ventilatory responses in adults with cystic fibrosis

**DOI:** 10.1186/s12931-020-01406-z

**Published:** 2020-06-01

**Authors:** Bradley S. Quon, Andrew H. Ramsook, Satvir S. Dhillon, Reid A. Mitchell, Kyle G. Boyle, Pearce G. Wilcox, Jordan A. Guenette

**Affiliations:** 1 Clinician-Scientist, UBC Centre for Heart Lung Innovation, Providence Health Care Research Institute, University of British Columbia, St. Paul’s Hospital, #166 – 1081 Burrard Street, Vancouver, BC V6Z 1Y6 Canada; 2 Division of Respiratory Medicine, Department of Medicine, University of British Columbia, Vancouver, BC Canada; 3 Department of Physical Therapy, University of British Columbia, Vancouver, BC Canada

## Abstract

**Rationale:**

Lumacaftor/ivacaftor (LUM/IVA) modestly improves lung function following 1 month of treatment but it is unknown if this translates into improvements in exercise endurance and exertional symptoms.

**Methods:**

Adult CF participants completed a symptom-limited constant load cycling test with simultaneous assessments of dyspnea and leg discomfort ratings pre- and 1 month post-initiation of LUM/IVA.

**Results:**

Endurance time, exertional dyspnea and leg discomfort ratings at submaximal exercise did not change significantly. There was a significant inverse correlation between changes in leg discomfort and endurance time (r = − 0.88; *p* = 0.009) following 1-month of LUM/IVA.

**Conclusions:**

Overall, 1-month of LUM/IVA did not increase endurance time or modify exertional dyspnea or leg discomfort ratings. However, individuals who experienced a reduction in leg discomfort following LUM/IVA had an improvement in endurance time. Future studies with a larger sample size are needed to verify these findings and to assess the long-term effects of LUM/IVA on exercise outcomes.

**Trial registration:**

ClinicalTrials.gov Identifier: NCT02821130. Registered July 1, 2016.

## Introduction

Exercise capacity is an important outcome parameter for individuals with cystic fibrosis (CF) and is predictive of survival [1, 2]. Cystic fibrosis transmembrane conductance regulator (CFTR) modulators, including lumacaftor/ivacaftor (Orkambi™) and tezacaftor-ivacaftor (Symdeko™), have been approved for use in CF individuals homozygous for F508del, but with modest effects on percent-predicted forced expiratory volume in 1 s (ppFEV_1_) [3, 4]. However, ppFEV_1_ does not fully capture how a patient functions, feels, or survives. As such, in this preliminary study, we evaluated short-term changes in exertional symptoms, exercise performance, and ventilatory responses in CF adults as part of a research protocol when lumacaftor/ivacaftor (LUM/IVA) was initiated as part of clinical care.

## Methods

We recruited 11 stable adult CF participants (≥19 years old) with ppFEV_1_ < 90% and homozygous for the F508del mutation in the CFTR gene and who were about to initiate LUM/IVA. To prevent patient-to-patient spread within the exercise laboratory of respiratory pathogens associated with poor outcomes in CF, individuals were excluded if they had prior growth of *Mycobacterium abscessus* subspecies or *Burkholderia cepacia* complex from respiratory culture within the past 2 years in compliance with our hospital’s infection prevention and control policies. Individuals were also excluded if they had any contraindication to exercise testing or if they had a medical condition (other than CF) that could contribute to dyspnea or exercise intolerance. This study received institutional ethical approval (University of British Columbia – Providence Health Care Research Ethics Board #H16–01164). Written informed consent was obtained from all participants.

This observational study involved 3 visits. The first visit was prior to LUM/IVA initiation and involved pulmonary function testing and a symptom-limited incremental cycle exercise test. Visits 2 (baseline; within 1 week prior to initiating LUM/IVA) and 3 (1-month post-initiation of full dose LUM/IVA) included questionnaires [St. George’s Respiratory Questionnaire (SQRG) and - International Physical Activity Questionnaire – Long Form (IPAQ-LF)], pulmonary function testing, and a symptom-limited constant load cycle (Ergoselect 200P; Ergoline GmbH, Bitz, Germany) exercise test at 80% of their peak incremental work rate. A fourth visit (3-month post-initiation of full dose LUM/IVA) was obtained in only 4 participants and therefore was not included in the present study. Exercise performance was measured as the duration of constant load cycling (i.e., endurance time). Standard cardiopulmonary variables were measured using a commercially available metabolic cart (TrueOne 2400™; ParvoMedics Inc., Utah, USA) and dyspnea and leg discomfort ratings were assessed at rest, every 2 minutes during exercise, and upon exercise cessation using the modified category-ratio 0–10 Borg scale [5]. Normality of data was tested with a Shapiro-Wilk test. A Wilcoxon-Signed rank test assessed differences between baseline and post-treatment variables. The correlation between the change in exercise time and the change in select cardiorespiratory variables were assessed with a Spearman correlation test. Data are presented as median (interquartile range).

Four of 11 participants had to be excluded from the analysis for the following reasons: 2 started treatment before their baseline visit; 1 did not show up for their 1-month follow-up visit; and 1 cultured *M. abscessus* from sputum culture between enrolment and baseline visits.

## Results

Descriptive characteristics of the study participants can be found in Table 1. Following 1 month of LUM/IVA treatment, ppFEV_1_ improved from 46 (18) to 49 (19) but this change was not statistically significant (*p* = 0.078) and there was no significant change in other pulmonary function measurements (Table 1). BMI increased from 20.2 (3.1) to 20.5 (3.0) kg/m^2^ (*p* = 0.031). Consistent with a CFTR modulating effect, sweat chloride (*n* = 5) decreased significantly from 103 (25) to 90 (18) mmol/L (*p* = 0.029). SGRQ total score and IPAQ-LF were also unchanged after treatment. One subject experienced a pulmonary exacerbation requiring IV antibiotics prior to visit 3. No other subjects had an exacerbation or required any other changes to their medications from baseline to visit 3.

**Table 1:** Clinical Characteristics and Static Pulmonary Function Measurements at Baseline and 1-month Post- LUM/IVA Treatment. Values are median (IQR)

	Baseline	Post-Treatment
Male:Female	3:4	
Age, years	29 (10)	30 (9)
Height, cm	164 (9)	164 (9)
Mass, kg	59.3 (9.5)	60.3 (9.1)
BMI, kg/m^2^	20.2 (3.1)	20.5 (3.0)*
SGRQ, total score	37.1 (17.8)	26.3 (16.9)
IPAQ-LF, MET-min/week	2910 (1676)	1754 (3322)
Sweat Chloride, mmol/L	103 (25)	90 (18)*
Pulmonary Function		
FEV_1,_ L	1.44 (0.22)	1.45 (0.45)
FEV_1_, %pred	46 (18)	49 (19)
FVC, L	3.17 (0.49)	3.11 (0.57)
FVC, %pred	73 (21)	69 (28)
FEV_1_/FVC, %	49.1 (10.8)	54.3 (10.3)
TLC, L	6.17 (1.91)	5.93 (2.10)
TLC, %pred	97 (17)	100 (27)
DL_CO,_ mL/mmHg/min	17.4 (6.5)	18.9 (6.6)
DL_CO_, %pred	78 (22)	90 (18)

Values are median (IQR). *Abbreviations:* BMI, body mass index; SGRQ, St. George’s Respiratory Questionnaire Score; IPAQ-LF, International Physical Activity Questionnaire – Long Form; FEV_1_, forced expired volume in 1 s; FVC, forced vital capacity; TLC, total lung capacity; DL_CO_, diffusing capacity of the lungs for carbon monoxide (data available in 6 participants);*p < 0.05

Table 2 shows the changes in select exercise variables. Six of the 7 participants improved their endurance time but this did not achieve statistical significance (*p* = 0.219), driven largely by one participant who had an 8.1 min decrease in endurance time. The poor exercise performance on the post treatment visit may have been related to the fact that the participant was being treated for a pulmonary exacerbation 2 weeks prior to their post-treatment exercise test.

**Table 2:** Exercise Responses Measured at Iso-time and Peak Exercise at Baseline and 1-month Post- LUM/IVA Treatment

Exercise	*Iso-time*		*Peak*	
	Baseline	Post-Treatment	Baseline	Post-Treatment
Exercise time, min	6.0 (4.0)	6.0 (4.0)	6.6 (5.6)	7.9 (3.6)
V̇O_2_, L/min	1.01 (0.61)	1.08 (0.57)	1.05 (0.61)	1.25 (0.52)
V̇O_2_, mL/kg/min	17.8 (9.9)	18.9 (7.8)	18.2 (9.5)	21.9 (7.9)
IRV, L	0.36 (0.37)	0.57 (0.44)	0.33 (0.39)	0.51 (0.37)
HR, beat/min	148 (25)	154 (23)	161 (23)	159 (15)
HR, %pred	79 (11)	83 (12)	81 (10)	85 (9)
V̇_E_, L/min	40.8 (16.1)	40.7 (17.8)	43.9 (20.2)	46.5 (13.0)
Fb, breath/min	35 (8)	36 (5)	39 (7)	42 (5)
V_T_, L	1.2 (0.2)	1.1 (0.4)	1.1 (0.3)	1.1 (0.3)
Dyspnea, Borg	6 (3)	4 (2)	8 (2)	7 (3)
Leg Discomfort, Borg	8 (3)	5 (4)	9 (4)	10 (2)
EELV, %TLC	72 (9)	71 (10)	73 (9)	72 (6)
EILV, %TLC	94 (4)	92 (8)	93 (4)	92 (3)
IC, L	1.66 (0.53)	1.84 (0.63)	1.52 (0.63)	1.68 (0.39)

Values are median (IQR). *Abbreviations:* iso-time, highest submaximal exercise time achieved by an individual on both baseline and post-treatment exercise tests; VO_2_, oxygen consumption; IRV, inspiratory reserve volume; HR, heart rate; V̇_E_, minute ventilation; Fb, breathing frequency; V_T_, tidal volume; EELV, end-expiratory lung volume; EILV, end-inspiratory lung volume, *p < 0.05

Breathing patterns and operating lung volumes were similar at baseline and following 1 month of full-dose LUM/IVA. There was also no significant change in Borg dyspnea ratings or leg discomfort ratings at submaximal exercise relative to baseline. However, 4 of 7 participants experienced a decrease in leg discomfort and overall there was a significant correlation between the reduction in leg discomfort at iso-time and the increase in endurance time (Spearman’s rho = − 0.88; *p* = 0.009).

## Discussion

In this study, we focused on the short-term effects of LUM/IVA. Longer-term studies of LUM/IVA are likely required to demonstrate effects on exercise capacity, particularly as exercise limitation in CF is complex and often related to peripheral muscle dysfunction and not ventilatory constraints, except in individuals with more advanced lung disease [6–8]. In support of this, participants who had an increase in their endurance time in our study experienced less leg discomfort but there was no relationship with changes in dyspnea. While an improvement in exercise time during an incremental exercise test was noted in a placebo controlled, cross-over study evaluating IVA in individuals with at least one copy of the G551D mutation treated for a similar period of 28 days, there was no difference in peak work rate or VO_2_ [9]. The clinical importance of a change in cycle time during an incremental exercise protocol with no change in peak work rate or VO_2_ is unclear. This study differed in design as we used a constant work rate protocol, which is considered to be a more sensitive and clinically relevant way of assessing exercise performance compared to incremental exercise protocols [10]. Nevertheless, the lack of change in peak VO_2_ in the setting of relatively large improvements in ppFEV_1_ in this previous study supports the notion that ventilatory limitation is likely not the sole driver of exercise limitation in CF. Therefore, other factors such as muscle dysfunction and deconditioning likely play more important roles. We did not evaluate muscle function in this study but there was no significant difference in self-reported physical activity levels based on the IPAQ-LF between baseline and 1-month post LUM/IVA initiation. In a longer-term study by Savi et al. evaluating the effects of LUM/IVA in 3 adults treated for at least 2 years, there was an increase in physical activity measurements and oxygen uptake values at anaerobic threshold and peak exercise on symptom-limited incremental exercise testing [11]. The precise physiological mechanisms underlying these improvements with LUM/IVA requires further investigation.

There are some limitations to this study that should be acknowledged. Firstly, recruitment was less than anticipated due to lack of public reimbursement for LUM/IVA. Secondly, this study was not placebo-controlled and therefore we cannot exclude a placebo effect for participants who reported less leg discomfort with exercise following initiation of LUM/IVA. Thirdly, physical exercise and performance of physiotherapy prior to and during the study were not controlled for and could have influenced exercise performance. While we did not capture details on physiotherapy before and after introduction of LUM/IVA, we did not observe a difference in physical activity levels. Finally, to reduce the burden related to frequent study visits, we did not include a familiarization visit for our constant work rate exercise test. While a learning effect could have contributed, at least in part, to the improvement in endurance time for some individuals, a learning effect was not observed in a prior study involving CF patients following repeated cycle ergometer exercise testing [12].

In conclusion, 1 month of LUM/IVA did not lead to an overall increase in endurance time or modify exertional dyspnea or leg discomfort ratings. However, there was a trend toward less leg discomfort at submaximal exercise, which correlated with increased endurance time. Given the importance of peripheral muscle dysfunction on exercise limitation in CF, the direct or indirect effects of CFTR modulators on muscle function should be evaluated in future studies.

## Data Availability

Available upon request.
